# Clinical Profile and Outcome of Patients Presenting With Acute-on-Chronic Liver Failure: A Single-Center Experience

**DOI:** 10.7759/cureus.64643

**Published:** 2024-07-16

**Authors:** Payila Aneesh, Alok Kumar Singh, Venkatesh Vaithiyam, Roshan George, Shabir Lone, Sanjeev Sachdeva, Ashok Dalal, Ajay Kumar, Barjesh C Sharma

**Affiliations:** 1 Gastroenterology, Govind Ballabh Pant Hospital, New Delhi, IND

**Keywords:** sofa score, organ failure, mortality, apasl, aclf

## Abstract

Background and aim: We aimed to study the etiologies and clinical profile and to describe the factors associated with mortality in acute-on-chronic liver failure (ACLF) patients at our center.

Methods: Patients meeting the Asian Pacific Association for the Study of the Liver (APASL) definition of ACLF were included. We studied etiologies and clinical profile and analyzed the factors associated with mortality in patients with ACLF. We also analyzed the mortality rates based on the number of organ failures and the grade of ACLF.

Results: 114 patients were included. Alcohol (82, 71.9%), drugs (22, 19.3%), and viral hepatitis (17, 14.9%) were the commonest precipitating factors of ACLF. The commonest cause of chronic disease was alcohol (83, 72.8%). Fifty-three (46.5%), 60 (52.6%), 44 (38.6%), 32 (28.1%), and 24 (21.1%) experienced renal, coagulation, cerebral, respiratory, and circulation failures, respectively. Overall, the in-hospital mortality rate stood at 54 (48.6%), with a median stay of eight days. Advanced hepatic encephalopathy and ventilator support independently predicted mortality. The Sequential Organ Failure Assessment (SOFA) score outperformed all other prognostic scores in predicting mortality in ACLF.

Conclusion: Alcohol was the most common precipitating factor for ACLF. The in-hospital mortality rate was 48.6%. Advanced hepatic encephalopathy and ventilator support independently predicted mortality. The SOFA score is a more accurate predictor of mortality in ACLF when compared to other prognostic scores.

## Introduction

Acute-on-chronic liver failure (ACLF) is a condition characterized by the sudden deterioration of liver function in patients with previously diagnosed or undiagnosed chronic liver disease. ACLF occurs when two liver insults happen at the same time [1]. The mortality rate of patients with ACLF has been stable over the previous two decades, with a rate exceeding 50% [2]. ACLF, as defined by the European Association for the Study of the Liver and the American Association for the Study of Liver Diseases (EASL-AASLD), refers to the sudden worsening of an existing chronic liver disease, typically caused by a specific triggering event, and is characterized by a higher risk of death within three months due to multiple organ failure [3]. According to the Asian Pacific Association for the Study of the Liver (APASL), ACLF is defined as a sudden liver injury that occurs in a patient with pre-existing diagnosed or undiagnosed chronic liver disease. It is characterized by jaundice (bilirubin levels above 5 mg/dl) and coagulopathy (international normalized ratio (INR) >1.5) and is further complicated by the presence of ascites and/or encephalopathy within four weeks [4]. The APASL definition includes both persons with cirrhosis and without cirrhosis, while the EASL-AASLD definition only applies to persons with cirrhosis. Recently, the World Health Organization proposed an alternative definition that merges the requirements of APASL and EASL-AASLD. This study aimed to investigate the causes, clinical spectrum, and factors associated with mortality in patients with ACLF at our facility. We also analyzed the mortality rates based on the number of organ failures and the grade of ACLF.

## Materials and methods

All patients with ACLF who met the definition provided by the APASL and were admitted to the Department of Gastroenterology at the Govind Ballabh Pant Institute of Post-Graduate Medical Education and Research in New Delhi between September 2018 and October 2023 were included in the study. We retrospectively gathered patient data from our database, which was originally maintained prospectively. In our analysis, we included patients who had non-hepatic acute insults and pre-existing decompensation, although they were excluded under the APASL-ACLF criterion. This decision was made because such criteria were followed in many other studies [5,6]. As our investigation was conducted retrospectively, ethical approval was waived.

Patients who fulfilled the APASL criteria and experienced either liver or non-liver insults were included, regardless of whether they had previously experienced decompensation. Patients who were pregnant and had disseminated malignancy, portal vein thrombosis, ambiguous diagnoses, and insufficient data were excluded from the study.

The presence of chronic liver disease was determined through a comprehensive assessment that included a thorough clinical history, physical examination, laboratory tests, and radiological findings such as abnormal liver texture, shrinkage of the liver, nodular liver outline, and signs of portal hypertension such as dilated portal vein, ascites, and splenomegaly [7]. Additionally, the diagnosis was confirmed through the endoscopic visualization of varices or by examining the liver tissue obtained through a biopsy, which revealed characteristic changes associated with cirrhosis. Prognostic scores such as Sequential Organ Failure Assessment (SOFA) [8], Chronic Liver Failure Consortium Acute on Chronic Liver Failure (CLIF-C ACLF) [9], Acute Physiology and Chronic Health Evaluation (APACHE II) [10], Child-Turcotte-Pugh (CTP) [11], and Model for End-Stage Liver Disease Sodium (MELD-Na) [12] were computed. Each case was assessed for potential precipitating factors, such as persistent alcohol consumption (alcohol consumption within 28 days prior to the onset of jaundice), ingestion of hepatotoxic drugs, infections caused by hepatotropic viruses (hepatitis A, B, D, and E), recent major surgery, sepsis, or variceal bleeding. Assessment for autoimmune hepatitis and Wilson's disease was also performed if the etiology of the chronic liver disease could not be determined. When multiple acute triggering events were present, they were considered multiple acute insults. Organ failures and grading of ACLF were defined as per the EASL-CLIF Consortium.

Standard care therapy was followed for all patients. Patients diagnosed with hepatitis B virus (HBV) or hepatitis C virus (HCV) were prescribed antiviral medications. Patients diagnosed with Wilson's disease were initiated on penicillamine and zinc supplementation, whereas those diagnosed with autoimmune hepatitis were initiated on steroid treatment. Renal replacement therapy was administered as needed. Patients with grade 3 ascites underwent a large-volume paracentesis. Patients with hepatic encephalopathy were administered anti-coma medicines, such as rifaximin and lactulose. The usual protocols are used to treat hepatorenal syndrome and spontaneous bacterial peritonitis. Endoscopic variceal ligation (EVL) was performed as required. The decision to start and adjust the dosage of antibiotics, as well as the determination of the necessity for ventilator support, was made by the treating clinicians based on the clinical situation. None of the patients underwent liver transplantation.

Quantitative data are expressed as either the average (mean) or the middle value (median). The proportions were used to represent categorical data. A univariate analysis was conducted to compare survivors and non-survivors. An independent t-test, or Mann-Whitney U-test, was used to analyze continuous data. The chi-squared test was used to analyze categorical variables. We used the Cox proportional hazards model to predict patient survival. A p-value less than 0.05 was used to assess statistical significance.

## Results

In total, 114 patients with ACLF were studied. The mean age was 39.11±10.77, with 93 (81.6%) males and 21 (18.4%) females. Ascites were present in 110/114 (96.49%) patients. Twenty-five (21.9%) patients had early hepatic encephalopathy (HE), and 43 (37.7%) had advanced HE. The baseline clinical presentation and biochemical characteristics of the 114 patients are presented in Table 1. 

**Table 1:** Baseline clinical and laboratory characteristics of patients with ACLF (n=114) AARC: Asian Pacific Association for the Study of Liver Acute on Chronic Liver Failure Research Consortium; ACLF: acute-on-chronic liver failure; AIH: autoimmune hepatitis; ALP: alkaline phosphatase; ALT: alanine transaminase; APACHE II: Acute Physiology and Chronic Health Evaluation; AST: aspartate transaminase; ATT: antitubercular therapy; CAM: complementary and alternative medicine; CLIF-C ACLF: Chronic Liver Failure Consortium Acute on Chronic Liver Failure; CTP: Child-Turcotte-Pugh; DF: Maddrey's discriminant function; DILI: drug-induced liver injury; GM-CSF: granulocyte monocyte colony-stimulating factor; gm/dl: gram per deciliter; HAV: hepatitis A virus; HBV: hepatitis B virus; HCC: hepatocellular carcinoma; HCV: hepatitis C virus; HEV: hepatitis E virus; HE: hepatic encephalopathy; INR: international normalized ratio; IQR: interquartile range; IU/L: international units per liter; MELD-Na: Model for End-Stage Liver Disease Sodium; meq/L: milliequivalents per liter; mg/dl: milligram per deciliter; mm^3^: cubic millimeter; n: sample size; SD: standard deviation; SOFA: Sequential Organ Failure Assessment; TLC: total leukocyte count; %: percentage Reference ranges: hemoglobin: males: 14-17 gm/dl; females: 12-16 gm/dl; total leukocyte count: 4000-10,000 cells per mm^3^; platelets: 1.5-3.5 lakhs per mm^3^; creatinine: 0.8-1.3 mg/dl; sodium: 136-145 meq/L; potassium: 3.5-5.0 meq/L; bilirubin: 0.3-1.0 mg/dl; AST: 10-40 IU/L; ALT: 10-40 IU/L; ALP: 30-120 IU/L; serum albumin: 3.5-5.5 gm/dl; INR: 0.8-1.2

Patient characteristics	All ACLF patients (n=114)
Age, mean±SD (years)	39.11±10.77
Males, n (%)	93 (81.6%)
Females, n (%)	21 (18.4%)
Ascites, n (%)	110 (96.49%)
Grade 1 ascites, n (%)	8 (7.01%)
Grade 2 ascites, n (%)	60 (52.63%)
Grade 3 ascites, n (%)	42 (36.8%)
No HE, n (%)	46 (40.4%)
Early HE (grades 1 and 2), n (%)	25 (21.9%)
Advanced HE (grades 3 and 4), n (%)	43 (37.7%)
Hemoglobin, gm/dl (mean±SD)	9.0±2.1
TLC, per mm^3^, median (IQR)	16200 (9350-22425)
Platelets, lakhs per mm^3^, median (IQR)	0.97 (0.57-1.46)
Creatinine (mg/dl), median (IQR)	1.8 (0.9-3.2)
Sodium, meq/L (mean±SD)	130±8.51
Potassium, meq/L (mean±SD)	4.27±0.83
Bilirubin, mg/dl (mean±SD)	20.44±9.59
AST, IU/L, median (IQR)	129 (91-197)
ALT, IU/L, median (IQR)	45 (29-72)
ALP, IU/L, median (IQR)	128 (97-183)
Serum albumin, gm/dl (mean±SD)	2.66±0.54
INR, median (IQR)	2.6 (2.0-3.4)
CLIF-C ACLF score (n=105) (mean±SD)	52.76±12.73
SOFA score (n=92) (mean±SD)	10.73±4.43
APACHE II score (n=94) (mean±SD)	17.68±7.86
DF (n=81) (mean±SD)	93.28±41.10
CTP A, n (%)	0
CTP B, n (%)	6 (5.3%)
CTP C, n (%)	108 (94.7%)
MELD-Na (n=114) (mean±SD)	33.62±6.12
AARC score (n=73) (mean±SD)	10.86±1.98
Etiology of acute insult	
Active alcoholism, n (%)	82 (71.9%)
Drug-induced liver injury, n (%)	22 (19.3%)
CAM, n (%)	20 (17.5%)
ATT, n (%)	2 (1.8%)
Viral infections (HAV, HBV, HEV), n (%)	17 (14.9%)
HEV, n (%)	10 (8.8%)
HBV, n (%)	5 (4.4%)
HAV, n (%)	1 (0.9%)
Combined HBV+HEV, n (%)	1 (0.9%)
Sepsis, n (%)	6 (5.3%)
AIH, n (%)	5 (4.4%)
Cryptogenic, n (%)	4 (3.5%)
Wilson's disease, n (%)	3 (2.6%)
Variceal bleed, n (%)	3 (2.6%)
Lupus hepatitis flare, n (%)	1 (0.9%)
HCC, n (%)	1 (0.9%)
Number of acute insults	
1, n (%)	80 (70.2%)
2, n (%)	31 (27.2%)
Acute insult type	
Hepatic insult, n (%)	109 (95.6%)
Extrahepatic insult, n (%)	9 (7.9%)
Both, n (%)	6 (5.3%)
Etiology of chronic liver disease	
Alcohol, n (%)	83 (72.8%)
Cryptogenic, n (%)	12 (10.52%)
HBV, n (%)	8 (7.0%)
AIH, n (%)	5 (4.4%)
Wilson's disease, n (%)	3 (2.6%)
Alcohol plus HCV, n (%)	1 (0.9%)
Secondary biliary cirrhosis, n (%)	1 (0.9%)
Lupus hepatitis, n (%)	1 (0.9%)
Prior decompensation, n (%)	20 (17.5%)
Comorbidities, n (%)	12 (10.5%)
Total hospital stay (days), median (IQR)	8 (6-14)
GM-CSF, n (%)	15 (13.2%)

The most frequent precipitating event was active alcoholism in 82 patients (71.9%), followed by drug-induced liver injury (DILI) and hepatotropic viral infections (hepatitis A, B, and E) in 22 (19.3%) and 17 (14.9%) patients, respectively. Sepsis and variceal bleeding were the most common extrahepatic precipitating events observed in six (5.3%) and three (2.6%) patients, respectively.

**Figure 1:**
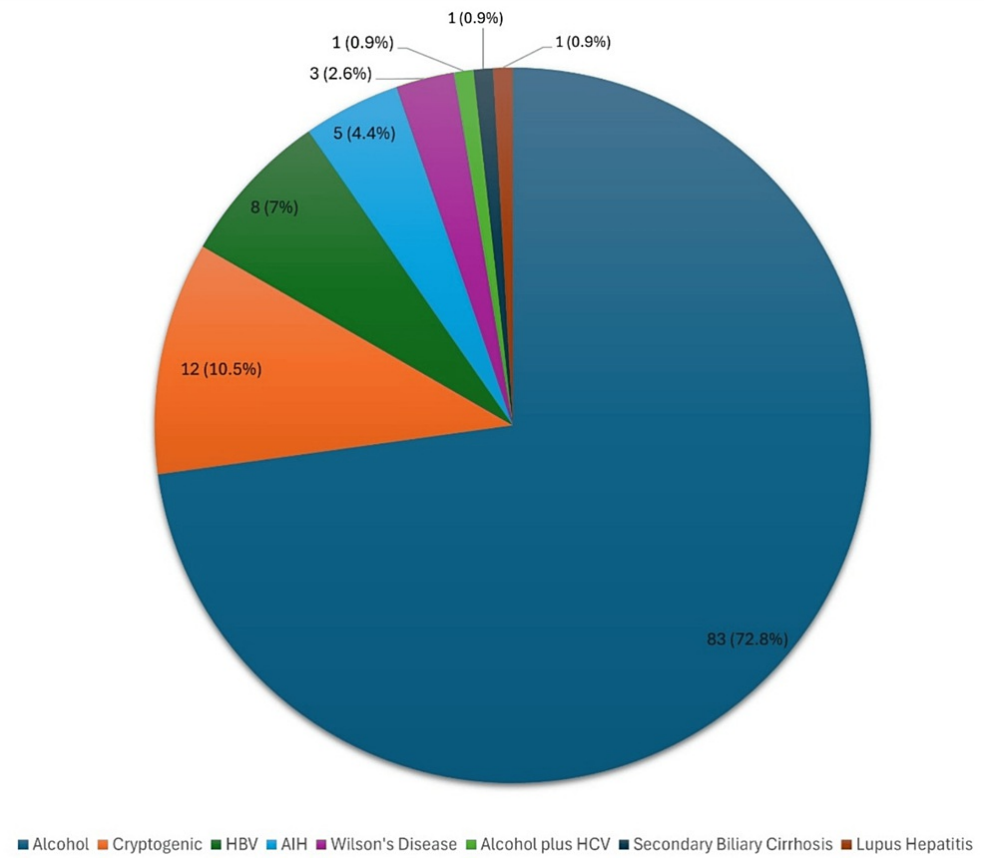
Etiologies of chronic liver disease in ACLF patients ACLF: acute-on-chronic liver failure; AIH: autoimmune hepatitis; HBV: hepatitis B virus; HCV: hepatitis C virus; %: percentage

Alcohol was the most common cause of chronic liver disease, seen in 83 (72.8%), followed by HBV seen in eight (7%). Five patients (4.4%) had autoimmune hepatitis, and three patients (2.6%) had Wilson's disease. Lupus hepatitis was identified in one patient (0.9%). Etiology (cryptogenic) was not found in 12 patients (10.52%) (Figure 1).

**Figure 2:**
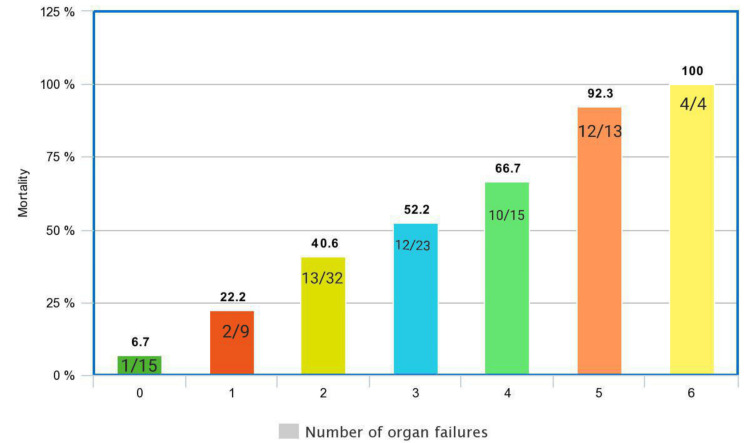
In-hospital mortality according to the number of organ failures (n=54) n: sample size; %: percentage

Of all 114 patients, liver failure was observed in 84 (73.7%), coagulation failure in 60 (52.6%), renal failure in 53 (46.5%), cerebral failure in 44 (38.6%), respiratory failure in 32 (28.1%), and circulatory failure in 24 (21.1%). Fifteen (13.2%) patients had no organ failure, whereas one, two, three, four, five, and six organ failures were recorded in 10 (8.8%), 33 (28.9%), 24 (21.1%), 15 (13.2%), 13 (11.4%), and four (3.5%) patients, respectively. Although baseline characteristics were assessed for 114 patients, the outcomes of three ACLF patients were not known; hence, survivor and non-survivor analyses were performed for only 111 patients. The mortality rate was 6.7% (1/15) in patients with no organ failure and 100% (4/4) in patients with six organ failure (Figure 2).

**Figure 3:**
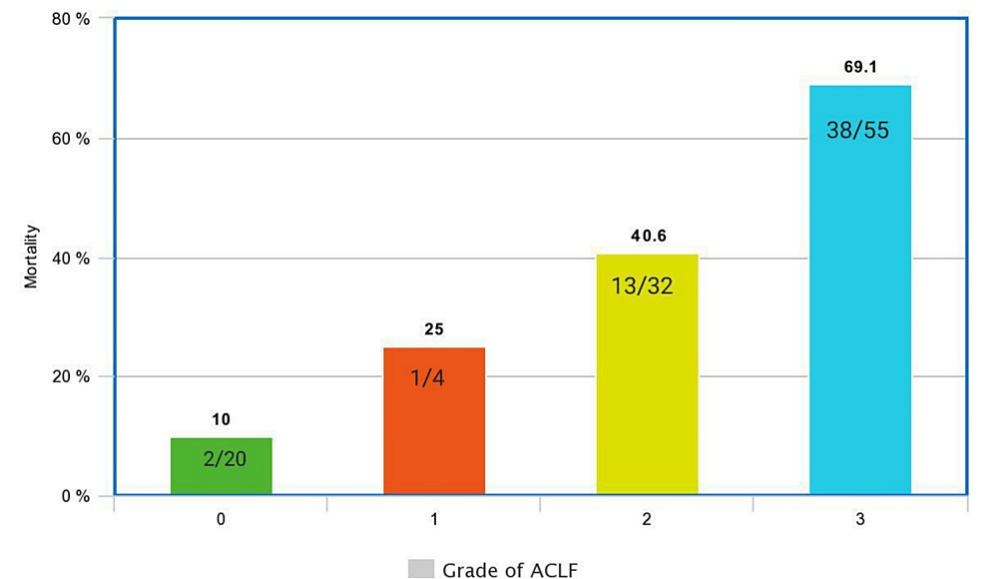
In-hospital mortality as per the grade of ACLF (n=54) ACLF: acute-on-chronic liver failure; n: sample size; %: percentage

Mortality rates varied according to the ACLF grades: ACLF 1 (25%), ACLF 2 (40.6%), and ACLF 3 (69.1%) (Figure 3).

Serum creatinine, serum bilirubin, advanced HE, and ventilator support needs were higher in the non-survivors. Statistically, all organ failures, except coagulation failure, were more frequent among non-survivors. Non-survivors had higher MELD-Na, SOFA, CLIF-C ACLF, AARC, and APACHE II scores (Table 2).

**Table 2:** Comparison of variables between survivors and non-survivors in patients with ACLF AARC: Asian Pacific Association for the Study of Liver Acute on Chronic Liver Failure Research Consortium; ACLF: acute-on-chronic liver failure; AIH: autoimmune hepatitis; ALP: alkaline phosphatase; ALT: alanine transaminase; APACHE II: Acute Physiology and Chronic Health Evaluation; AST: aspartate transaminase; CLIF-C ACLF: Chronic Liver Failure Consortium Acute on Chronic Liver Failure; CTP: Child-Turcotte-Pugh; DF: Maddrey's discriminant function; DILI: drug-induced liver injury; gm/dl: gram per deciliter; HAV: hepatitis A virus; HBV: hepatitis B virus; HCC: hepatocellular carcinoma; HCV: hepatitis C virus; HEV: hepatitis E virus; HE: hepatic encephalopathy; INR: international normalized ratio; IQR: interquartile range; IU/L: international units per liter; MELD-Na: Model for End-Stage Liver Disease Sodium; meq/L: milliequivalents per liter; mg/dl: milligram per deciliter; mm^3^: cubic millimeter; SD: standard deviation; SOFA: Sequential Organ Failure Assessment; TLC: total leukocyte count; n: sample size; %: percentage Reference ranges: hemoglobin: males: 14-17 gm/dl; females: 12-16 gm/dl; total leukocyte count: 4000-10,000 cells per mm^3^; platelets: 1.5-3.5 lakhs per mm^3^; creatinine: 0.8-1.3 mg/dl; sodium: 136-145 meq/L; potassium: 3.5-5.0 meq/L; bilirubin: 0.3-1.0 mg/dl; AST: 10-40 IU/L; ALT: 10-40 IU/L; ALP: 30-120 IU/L; serum albumin: 3.5-5.5 gm/dl; INR: 0.8-1.2 Independent t-test and Mann-Whitney U-test were used for analyzing continuous variables. Chi-squared test was used for analyzing categorical variables

Variable	Non-survivors (n=54)	Survivors (n=57)	P-value
Age (years), (mean±SD)	40.43±10.55	37.35±10.56	0.128
Sex (male:female)	42:12	49:8	0.262
Comorbidities, n (%)	4 (7.4%)	7 (12.3%)	0.390
Hemoglobin, gm/dl, (mean±SD)	8.70±1.92	9.15±2.22	0.257
TLC, per mm^3^, median (IQR)	18450 (9575-25775)	15200 (8000-20600)	0.601
Platelet, lakhs per mm^3^, median (IQR)	0.99 (0.65-1.48)	0.90 (0.49-1.46)	0.633
Total bilirubin, mg/dl, (mean±SD)	22.85±10.22	17.75±8.21	0.004
AST, IU/L, median (IQR)	140 (101-202)	123 (75-190)	0.459
ALT, IU/L, median (IQR)	45 (34-76)	45 (28-71)	0.165
ALP, IU/L, median (IQR)	126 (94-188)	132 (100-183)	0.563
Serum albumin, gm/dl, (mean±SD)	2.64±0.58	2.67±0.50	0.76
Creatinine, mg/dl, median (IQR)	2.55 (1.17-3.42)	1.40 (0.80-2.62)	0.008
Sodium, meq/L, (mean±SD)	131.22±10.07	128.44±6.51	0.08
Potassium, meq/L, (mean±SD)	4.27±0.94	4.27±0.72	0.99
INR, (mean±SD)	3.24±1.71	2.70±1.24	0.06
MELD-Na, (mean±SD)	35.94±5.91	31.16±4.80	0.001
DF, (mean±SD)	93.02±35.21 (n=38)	94.30±46.37 (n=42)	0.891
CLIF-C ACLF score, (mean±SD)	57.67±14.03 (n=49)	48.28±9.64 (n=53)	0.001
SOFA score, (mean±SD)	12.60±4.56 (n=50)	8.56±3.07 (n=41)	0.001
APACHE II score, (mean±SD)	20.96±7.66 (n=49)	14.43±6.14 (n=44)	0.001
AARC score, (mean±SD)	11.50±1.88 (n=38)	10.24±1.86 (n=34)	0.006
Ventilator support, n (%)	25 (46.3%)	0	<0.001
Total hospital stay (days), median (IQR)	8.0 (5.5-14.0) (n=54)	9.0 (6.0-15.0) (n=55)	0.888
Early HE (grades 1 and 2), n (%)	10 (18.5%)	13 (22.8%)	0.577
Advanced HE (grades 3 and 4), n (%)	32 (59.3%)	11 (19.3%)	<0.001
Etiology of acute insult			
Alcohol, n (%)	37 (68.5%)	44 (77.2%)	0.304
DILI, n (%)	12 (22.2%)	10 (17.5%)	0.537
Viral, (HAV, HBV, HEV), n (%)	6 (11.1%)	9 (15.8%)	0.471
Sepsis, n (%)	4 (7.4%)	2 (3.5%)	0.364
AIH, n (%)	4 (7.4%)	1 (1.8%)	0.151
Cryptogenic, n (%)	2 (3.7%)	2 (3.5%)	0.956
Wilson's disease, n (%)	1 (1.9%)	2 (3.5%)	0.591
Variceal bleed, n (%)	0	3 (5.3%)	0.087
Lupus hepatitis flare, n (%)	1 (1.9%)	0	0.302
HCC, n (%)	1 (1.9%)	0	0.302
Number of acute insults			
1, n (%)	38 (71.7%)	39 (70.9%)	0.928
2, n (%)	15 (28.3%)	16 (29.1%)	0.928
Hepatic acute insult, n (%)	51 (94.4%)	55 (96.5%)	0.403
Extrahepatic acute insult, n (%)	4 (7.4%)	5 (8.8%)	0.455
Chronic insult etiology			0.582
Alcohol, n (%)	38 (70.4%)	44 (77.2%)	
Cryptogenic, n (%)	6 (11.1%)	6 (10.5%)	
HBV, n (%)	3 (5.6%)	3 (5.3%)	
AIH, n (%)	4 (7.4%)	1 (1.8%)	
Wilson's disease, n (%)	1 (1.9%)	2 (3.5%)	
Alcohol plus HCV, n (%)	0	1 (1.8%)	
Secondary biliary cirrhosis, n (%)	1 (1.9%)	0	
Lupus hepatitis, n (%)	1 (1.9%)	0	
Organ failure			
Liver failure, n (%)	45 (83.3%)	36 (63.2%)	0.017
Coagulation failure, n (%)	30 (55.6%)	28 (49.1%)	0.498
Renal failure, n (%)	31 (57.4%)	21 (36.8%)	0.03
Cerebral failure, n (%)	33 (61.1%)	11 (19.3%)	<0.001
Respiratory failure, n (%)	31 (57.4%)	1 (1.8%)	<0.001
Circulation failure, n (%)	18 (33.3%)	6 (10.5%)	0.004
CLIF-C ACLF grading			<0.001
No ACLF, n (%)	2 (3.7%)	18 (31.6%)	
Grade 1 ACLF, n (%)	1 (1.9%)	3 (5.3%)	
Grade 2 ACLF, n (%)	13 (24.1%)	19 (33.3%)	
Grade 3 ACLF, n (%)	38 (70.4%)	17 (29.8%)	
AARC grading	(n=38)	(n=32)	0.02
Grade 1 AARC, n (%)	1 (2.6%)	2 (6.3%)	
Grade 2 AARC, n (%)	9 (23.7%)	17 (53.1%)	
Grade 3 AARC, n (%)	28 (73.7%)	13 (40.6%)	

The Cox proportional hazards model showed advanced HE and ventilator support independently predicted mortality, with hazard ratios (HR) of 2.762 (95% confidence interval (CI) 1.470-5.187, p=0.002) and 2.225 (95% CI 1.250-3.961, p=0.007), respectively (Table 3).

**Table 3:** Regression analysis using Cox proportional hazards model to predict the in-hospital survival of ACLF patients ACLF: acute-on-chronic liver failure; CI: confidence interval; HE: hepatic encephalopathy; HR: hazard ratio

Variable	P-value	HR	95% CI for HR	
			Lower	Upper
Advanced HE	0.002	2.762	1.470	5.187
Ventilator requirement	0.007	2.225	1.250	3.961

**Figure 4:**
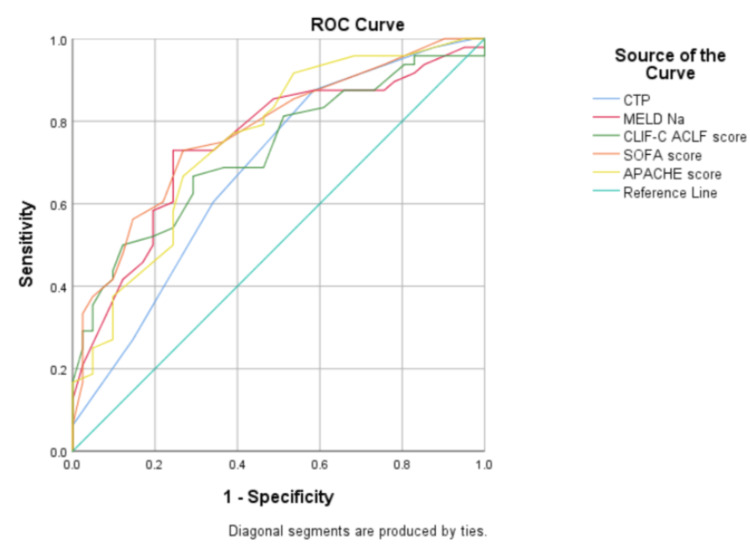
ROC curves for various prognostic indices: CTP, MELD-Na, CLIF-C ACLF, SOFA, and APACHE II scores APACHE II: Acute Physiology and Chronic Health Evaluation; CLIF-C ACLF: Chronic Liver Failure Consortium Acute on Chronic Liver Failure; CTP: Child-Turcotte-Pugh; MELD-Na: Model for End-Stage Liver Disease Sodium; ROC curve: receiver operating characteristic curve; SOFA: Sequential Organ Failure Assessment

The area under receiver operating characteristic curve (AUROC) for various prognostic indices (CTP, MELD-Na, SOFA score, CLIF-C ACLF score, and APACHE II score) were 0.682 (0.571-0.794), 0.747 (0.644-0.850), 0.775 (0.679-0.871), 0.726 (0.622-0.831), and 0.754 (0.653-0.855) (Figure 4).

## Discussion

ACLF can be reversed if the acute inciting event has been resolved. The acute triggering events of ACLF may originate from the liver itself (hepatic) or from outside the liver (extrahepatic). Continuous alcohol use was the most frequent cause of ACLF in our study, accounting for 71.9% of the cases. This finding is consistent with previous Indian studies conducted by Pati et al. [13] and Shalimar et al. [6]. In our investigation, bacterial infections and variceal bleeding constituted 5.3% and 2.6% of cases, respectively. These results closely align with the data observed in the western region. The CANONIC investigation, which involved 303 patients with ACLF, found that the most frequent causes of acute insults were bacterial infections (32.6%), alcoholism (24.5%), and gastrointestinal hemorrhage (13.2%). However, no acute insult was identified in 43.6% of the patients [14]. Our investigation found that viral hepatitis was a contributing factor for 14.9% of the patients. Similar results were observed in an earlier Indian study conducted by Shalimar et al. (21.4%) and Pati et al. (13.7%) [6,13]. The higher prevalence of viral hepatitis as a cause of acute insult for ACLF in India is likely attributed to the endemic presence of hepatitis A virus (HAV) and hepatitis E virus (HEV) in the country. In this study, we also identified a case of systemic lupus erythematosus presenting for the first time as severe liver injury leading to ACLF, which is quite rarely reported in previous literature. The most prevalent cause of cirrhosis in our analysis was alcohol consumption, which accounted for 72.8% of the cases. This finding is consistent with those of other investigations, such as the CANONIC study [14] and Duseja et al. [15]. In addition, approximately half of the individuals (45%) with active alcoholism died in our study. This underscores the significant burden of alcoholic liver disease in our nation, emphasizing the need to address this escalating threat to public health. According to the CANONIC study, the presence of organ failure is necessary to define an ACLF. The CANONIC study [14] found that the mortality rates at 28 and 90 days in patients with ACLF were directly correlated with the severity of ACLF. Our study found that the in-hospital mortality rate increased from 25% to 69% when the ACLF grade went from 1 to 3. This trend has also been observed in other studies conducted in India [6]. In our investigation, the occurrence of liver failure was documented at a rate of 73.7%, whereas respiratory failure was reported at a rate of 28.1%. In comparison, the CANONIC trial reported liver failure rates of 43.6% and respiratory failure rates of 9.2%. The greater prevalence of organ failure in our study suggests that the included patients were in a more severe state of illness. The overall fatality rate in our study was 48.6%, with a median hospital stay of eight days. This rate is similar to that reported in the study conducted by Shalimar et al. [6]. Non-survivors had significantly elevated levels of serum bilirubin and serum creatinine compared with survivors. The non-survivors exhibited better prognostic scores. Mortality was independently predicted using advanced HE and ventilator support. Additionally, we found that the SOFA score outperformed all other prognostic indicators in accurately predicting ACLF mortality.

Strengths of our study include a large sample size and comprehensive evaluation of demographic, clinical, laboratory, and prognostic factors of patients with ACLF. However, our study was constrained by its retrospective approach, which might cause potential selection bias, which in turn might influence the conclusions of our study. Also, we did not assess mortality rates at 28 days or in the long term. We exclusively assessed mortality in the hospital setting. Patients were diagnosed with ACLF based on the diagnosis provided by the APASL. However, for the purpose of this investigation, organ failure was characterized according to the criteria established in the CANONIC study.

## Conclusions

Alcohol was the most common precipitating factor for ACLF (71.9%) and also the commonest cause of underlying chronic liver disease (72.8%) in our study. Total in-hospital mortality was 48.6%. Mortality proportionately increased with an increase in the number of organ failures (6.7% with no organ failure to 100% with six organ failures) and an increase in the grade of ACLF (25% with grade 1 ACLF to 69.1% with grade 3 ACLF). Advanced hepatic encephalopathy (HR: 2.762) and the need for a ventilator (HR: 2.225) independently predicted mortality in ACLF patients. The SOFA score (AUROC: 0.775) outperformed all other prognostic scores in predicting mortality in ACLF patients.
